# *Galactinol synthase 1* improves cucumber performance under cold stress by enhancing assimilate translocation

**DOI:** 10.1093/hr/uhab063

**Published:** 2022-01-20

**Authors:** Haibo Dai, Zihui Zhu, Zhenguang Wang, Zhiping Zhang, Weiwen Kong, Minmin Miao

**Affiliations:** 1 College of Horticulture and Plant Protection, Yangzhou University, Yangzhou 225009, China; 2 Joint International Research Laboratory of Agriculture and Agri-Product Safety of Ministry of Education of China, Yangzhou University, Yangzhou 225009, China; 3 Key Laboratory of Plant Functional Genomics of the Ministry of Education/Jiangsu Key Laboratory of Crop Genomics and Molecular Breeding, Yangzhou University, Yangzhou 225009, China

## Abstract

Cucumber (*Cucumis sativus* L.) predominantly translocates raffinose family oligosaccharides (RFOs) in the phloem and accumulates RFOs in leaves. Galactinol synthase (GolS) catalyzes the critical step of RFO biosynthesis, and determining the functional diversity of multiple GolS isoforms in cucumber is of scientific significance. In this study, we found that all four isoforms of *CsGolS* in the cucumber genome were upregulated by different abiotic stresses. β-Glucuronidase staining and tissue separation experiments suggested that *CsGolS1* is expressed in vascular tissues, whereas the other three *CsGolS*s are located in mesophyll cells. Further investigation indicates that *CsGolS1* plays double roles in both assimilate loading and stress response in minor veins, which could increase the RFO concentration in the phloem sap and then improve assimilate transport under adverse conditions. Cold-induced minor vein-specific overexpression of *CsGolS1* enhanced the assimilate translocation efficiency and accelerated the growth rates of sink leaves, fruits, and whole plants under cold stress. Finally, our results demonstrate a previously unknown response to adverse environments and provide a potential biotechnological strategy to improve the stress resistance of cucumber.

## Introduction

Galactinol synthases (GolS; EC 2.4.1.123) belong to the eukaryotic glycosyltransferase family (EC 2.4.x.y.) and catalyze the reaction of galactinol biosynthesis from *myo*-inositol and UDP-galactose [1]. Galactinol is an important molecule in plant defense itself and a galactosyl donor to generate raffinose, stachyose, verbascose, and larger raffinose family oligosaccharides (RFOs) [1, 2]. To date, the biosynthetic pathway of galactinol and RFOs catalyzed by GolS has only been found in high plants [1].

Plant genomes typically contain multiple copies of *GolS*s. Specifically, 7, 7, 8, 8, 20, and 9 *GolS*s have been identified in the genomes of arabidopsis (*Arabidopsis thaliana*), sesame (*Sesamum indicum*), apple (*Malus* × *domestica*), cassava (*Manihot esculenta*), rapeseed (*Brassica napus*), and tobacco (*Nicotiana tabacum*), respectively [3–7]. Phylogenetic analyses have revealed that these plant *GolS*s may have evolved from an ancestral fungal sequence and that multiple genome duplication and loss events occurred during evolution [1, 5]. The functional divergence of multiple GolS isoforms has been found in previously investigated plant species. Because GolS catalyzes the first step of RFO biosynthesis, the biological functions of GolSs are closely related to these oligosaccharides. RFOs accumulate in plant tissues under exposure to abiotic stresses and function as osmolytes or antioxidants. In addition, RFOs play roles in carbon storage and transport [8]. Therefore, *GolS*s in plants respond to different abiotic and biotic stresses and related phytohormones [3, 9], are expressed in maturing seeds and other storage organs [7, 10], and are transcribed in minor veins to synthesize RFOs for transport [11].

Genomics research on Cucurbitaceae has achieved great advances in recent years [12–19]. A genome-wide screening of cucumber (*Cucumis sativus*), melon, watermelon (*Citrullus lanatus*), pumpkin (*Cucurbita*), *Momordica charantia*, *Lagenaria siceraria*, and *Benincasa hispida* revealed that four (all investigated species except *Cucurbita*) or five (*Cucurbita*) *GolS* isoforms also exist in Cucurbitaceae genomes (Supplementary Fig. S1). Cucurbits are generally acknowledged as RFO-translocating species [20]. Thus, in each cucurbit genome, at least one *GolS* should be responsible for RFO synthesis in the intermediate cells (ICs) of minor veins and likely plays a key role in the so-called polymer trapping loading mechanism [21–23]. *CmGolS1* has been proven to play this role in assimilate loading in melon [11]. Cucumber *CsGolS1* and melon *CmGolS1* are orthologous genes with very high homology (only three amino acid differences). Thus, *CsGolS1* should play a role in cucumber that is similar to that of *CmGolS1* in melon. The function of *CsGolS1* in assimilate loading was also suggested by Ma *et al*. [24]. However, Kim *et al*. [25] reported that *CsGolS1* also responds to *Pseudomonas chlororaphis* as a signaling molecule to induce systemic resistance, which indicates that *CsGolS1* or *CmGolS1* may play multiple roles in both assimilate loading and the response to pathogens. In addition, RFOs accumulate in abiotically stressed organs and act as protective molecules in cucurbits [26]. Therefore, an investigation of how assimilate translocation is affected when minor vein-specific GolS is regulated by abiotic or biotic stresses may reveal new physiological functions of the GolS. In this study, cucumber was selected as the representative species to investigate the tissue-specific and stress-induced expression pattern of its *GolS*s because we have accumulated abundant knowledge on RFO metabolism in this worldwide-important vegetable [27–30]. The results indicate that *CsGolS1* plays double roles in both assimilate loading and the cold response in leaf vascular tissues and could improve assimilate transport under adverse conditions.

## Results

### Raffinose family oligosaccharide biosynthesis-related gene expression, enzyme activity, and sugar contents regulated by abiotic stresses in cucumber leaves

**Figure 1:**
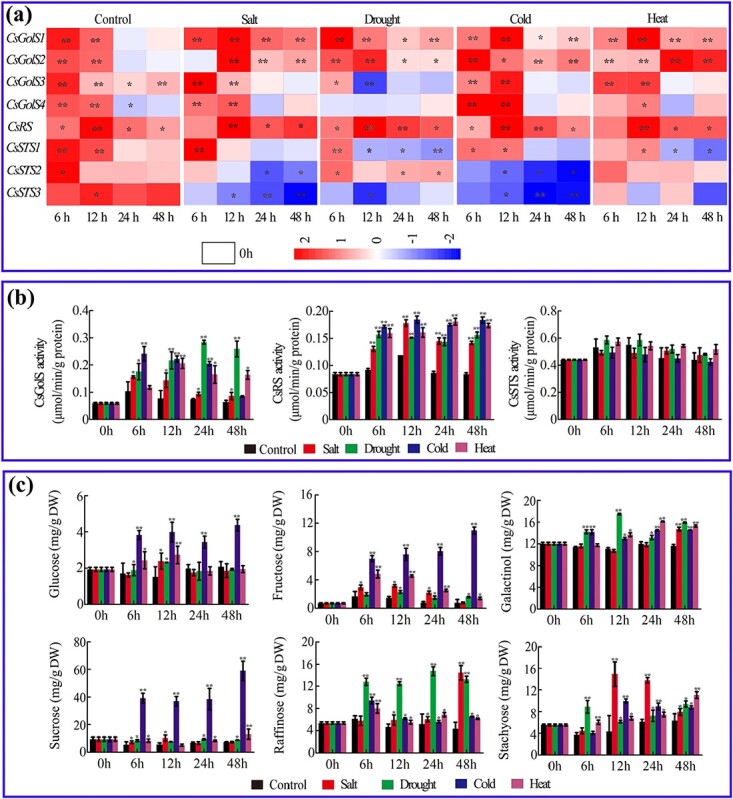
RFO biosynthesis-related gene expression (**a**), enzyme activity (**b**), and sugar contents (**c**) regulated by different stresses in cucumber source leaves (second leaf from the bottom of seedlings at the five-leaf stage). Enzyme activity is expressed as micromoles of product formed per minute per gram of protein, and the sugar contents estimated with the HPLC method are shown based on dry weight (DW). The data are shown as mean ± standard deviation (*n* = 5). **a** A heatmap of the relative gene expression level data identified by qRT–PCR (Supplementary Table S1). The 0-hour data for each gene were used for normalization; the value legend shows the fold difference in gene expression compared with 0 hours. The asterisks indicate significant differences between the treatments and the control at the same time point in (**b**) and (**c**) (Duncan’s test, ^*^P < .05, ^**^P < .01).

The expression patterns of four *CsGolS*s under different stresses are shown in Fig. 1a. The results indicate that all four *CsGolS*s responded to salt, drought, cold, and heat stress. Notably, different *CsGolS* isoforms responded to the same stress, even though the response times varied. For instance, all four *CsGolS*s were induced by cold stress. However, increases in the mRNAs of *CsGolS1* and *CsGolS2* were observed throughout the treatment (0–48 hours), whereas changes in the expression of *CsGolS3* and *CsGolS4* were only found within the first 12 hours. Similar phenomena were also observed with heat and salt treatments. The activities of cucumber CsGolS were also upregulated under all four types of abiotic stresses (Fig. 1b).

To understand the effects of stresses and GolS alterations on RFO biosynthesis, the expression and enzyme activity of two other important genes in the RFO biosynthesis pathway, raffinose synthase (*CsRS*) and stachyose synthase (*CsSTS*), and the sugar contents in cucumber leaves under exposure to different stresses were investigated. As shown in Fig. 1a and b, both the mRNA level and the enzyme activity of CsRS (EC 2.4.1.82) were upregulated under multiple stresses. Unexpectedly, a decrease in the mRNA abundance of *CsSTS*s was observed at most time points during the treatments. We also found that the enzyme activity of CsSTS (EC 2.4.1.67) decreased after exposure to cold stress (Fig. 1a and b). All the stress treatments induced the accumulation of most tested sugars at some time points. The fluctuation patterns of the contents of specific sugars under the different stresses were distinctive (Fig. 1c). We noted that the enzyme activities measured in this study were not always proportional to their metabolic substrates or products (for instance, the stachyose contents increased without any increase in CsSTS activity), which indicated the potential involvement of other factors, such as upstream and downstream metabolism and the tissue-specific compartments of these mRNAs, enzymes and sugars.

### Tissue-specific localization of four *CsGolS*s

**Figure 2:**
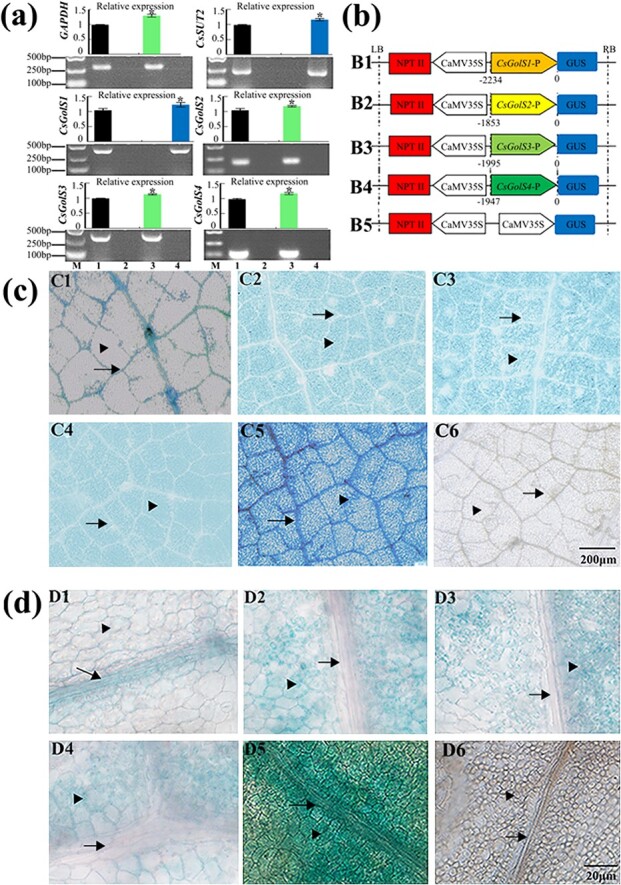
Tissue-specific localization of four *CsGolS* transcripts. Source leaves from seedlings at the five-leaf stage were used. **a** Tissue-specific expression of four *CsGolS* transcripts. RNA was extracted from total leaves (lane 1), the mesophyll fraction (lane 3), and the vascular fraction (lane 4) for qRT–PCR (histogram) and RT–PCR (electrophoretogram) analyses. To prepare the negative control, H_2_O was added to the reaction mix instead of cDNA (lane 2). Cucumber glyceraldehyde-3-phosphate dehydrogenase (*CsGAPDH*, NM_001305758.1) was used as a mesophyll-located marker gene, and cucumber sucrose transporter 2 (*CsSUT2*, NM_001305689) was used as a vascular-located marker gene. **b** Diagram of *CaMV35s*::*GUS* or *pCsGolS*s::*GUS* constructs used for transformation. RB, T-DNA right border; LB, left border. **c**, **d** Staining patterns of cucumber source leaves transformed with the constructs shown in **b***.* 1, *pCsGolS1*::*GUS*; 2, *pCsGolS2*::*GUS*; 3, *pCsGols3*::*GUS*; 4, *pCsGols4*::*GUS*; 5, *CaMV35s*::*GUS* (positive control); 6, WT (negative control). The data are shown as means ± standard deviation (*n* = 5). The asterisks in (**a**) indicate significant differences between leaves and other fractions (Duncan’s test, *P < .05). In (**c**) and (**d**) the scale bars represent 200 μm (**c**) and 20 μm (**d**). The arrows indicate the minor vein and the arrowheads indicate mesophyll cells.

To identify the tissue-specific expression patterns of the four *CsGolS*s, mechanical separation and reporter gene experiments were performed. As shown in Fig. 2a, the cucumber glyceraldehyde-3-phosphate dehydrogenase (*CsGAPDH*, NM_001305758.1, a mesophyll-located marker gene [31]) and sucrose transporter 2 (*CsSUT2*, NM_001305689, a vascular-located marker gene [24]) genes were exclusively expressed in the mesophyll and vein fractions, respectively, which indicated that the methods used for the mechanical separation of different leaf tissues used in this study are reliable. The results suggested that *CsGolS1* was expressed in the vein and that the other three *CsGolS* mRNAs were located in the mesophyll tissue. In the β-glucuronidase (GUS) staining assay, five, three, seven, and eight *pCsGolS*s::*GUS*-independent transformed plants were obtained to analyze the promoters of *CsGolS1*, *CsGolS2*, *CsGolS3*, and *CsGolS4*, respectively, and six *CaMV35S*::*GUS*-independent transformed plants were used as positive controls. A histochemical analysis of the leaves of these transformants was performed, and consistent GUS localization patterns were observed among the plants transformed with the same constructs (Supplementary Fig. S2). The typical staining patterns of each *pCsGolS*s::*GUS* T1 transformant are shown in Fig. 2b–d, and the results further confirm the tissue-specific expression patterns of the four *CsGolS*s.

### Tissue-specific characterization of gene expression, enzyme activity, and sugar content in cucumber leaves under different stresses

**Figure 3:**
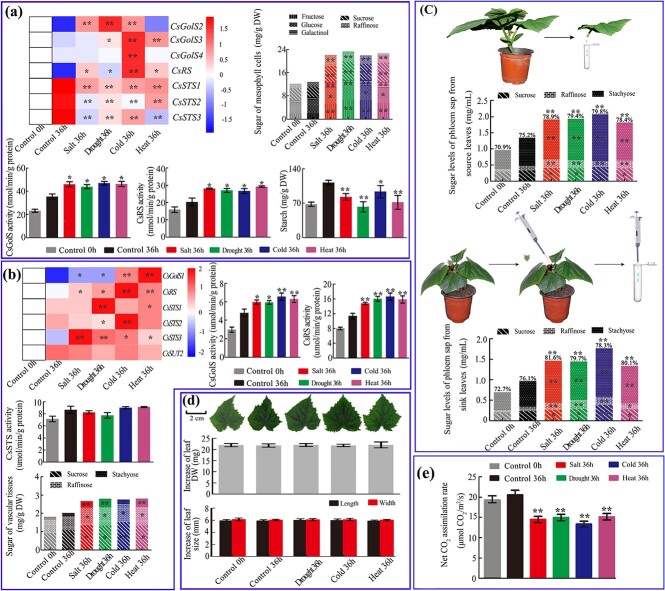
Tissue-specific characterization of gene expression, enzyme activity, and sugar content in cucumber leaves reveals a possible strategy used by cucumber to adapt to abiotic stresses. Seedlings at the five-leaf stage were used. **a** Mesophyll tissues from source leaves. **b** Vascular tissues from source leaves. **c** Phloem sap; the percentages on the columns indicate the ratio of RFOs to total sugars. **d** Increases in the dry weight and size of sink leaves (first unfolded leaf from bottom) after 0–36 hours of the treatments. **e** Net CO_2_ assimilation rates of the source leaves. Enzyme activity is expressed as micromoles of product formed per minute per gram of protein, and the sugar contents estimated with the HPLC method are shown based on the dry weight. The data are shown as mean ± standard deviation (*n* = 5 in **a**, **b**, and **c** and *n* = 15 in **d** and **e**). In the heatmaps, the expression level at 0 hour (white color) of each gene was used for normalization. The value legend shows the fold difference in gene expression compared with the control. The asterisks indicate significant differences between the treatments and the control at 36 hours (Duncan’s test, ^*^P < .05, ^**^P < .01).

A tissue localization study of four *CsGolS*s indicated that RFOs may be synthesized in both mesophyll and vein tissues of cucumber leaves (Fig. 2). To test this hypothesis, mesophyll and vein tissues were physically separated, and the gene expression profiles, enzyme activity, and sugar content in each fraction after 0 and 36 hours of the treatments were characterized. As shown in Fig. 3a, three mesophyll-located *CsGolS*s and *CsRS* and their enzyme activities were upregulated in mesophyll tissues by at least one abiotic stress treatment. Stress decreased the expression of *CsSTS*s in mesophyll tissues (Fig. 3a), and no CsSTS activity was detected (data not shown). In vein tissues, the expression of *CsGolS1*, *CsRS*, and *CsSTS*s and the activities of CsGolS and CsRS were upregulated by stress, whereas no notable change in CsSTS activity was found between the vascular tissues of stressed and control plants (Fig. 3b). Among all tested sugars, stachyose was not present in mesophyll tissues, whereas stachyose, sucrose, and raffinose were identified as three dominant sugars in the vascular tissues and phloem sap from petioles (Fig. 3a–c). Four abiotic stresses induced significant increases in most detectable sugars and the total soluble sugar content in mesophyll tissues, vascular tissues, and phloem sap. We further calculated the proportion of RFOs among the total sugars and found that the ratio of RFOs to total sugars in the phloem sap of source leaves also increased with all abiotic stresses, which indicated that the thicker phloem sap was not due to specific stress effects but rather to enhanced RFO synthesis in ICs (Fig. 3c). In combination with the data shown in Fig. 2, these results indicate that under stress conditions, cucumber *CsGolS1* is responsible for increasing the level of RFOs in the phloem, whereas the other three *CsGolS*s are responsible for raffinose accumulation in mesophyll cells. In addition to ‘polymer trap’ loading, cucumber also utilizes an apoplastic loading pathway in which *CsSUT2* plays a critical role [24]. To investigate whether this IC-specific transport protein contributes to the increase in sucrose level in the phloem sap of the source leaf, we further investigated the *CsSUT2* mRNA level. However, no change in *CsSUT2* expression was found after stress treatments (Fig. 3b).

The accumulation of RFOs in leaf tissues under abiotic stresses has been widely documented [3, 9]. However, the enrichment of soluble sugars and RFOs in phloem sap after stress has rarely been reported [32]. The assimilate translocation velocity within the phloem is often downregulated by abiotic stresses such as cold [32]. However, the similar growth rate of young leaves (Fig. 3d) observed under normal and stress conditions suggested that the translocation efficiency was not obviously affected by the stresses applied in our study. We deduced that the higher concentration of sugars, particularly RFOs, in sieve tubes may improve the assimilate translocation efficiency and offset the decrease in the transportation velocity induced by the stress treatments. To test this hypothesis, the sugar composition in the phloem sap of the sink leaf petiole was further analyzed. As shown in Fig. 3c, both the total sugar contents and the proportion of RFOs increased in the phloem sap of young leaves after exposure to stress. These results suggested that accelerating the assimilate translocation rate by the accumulation of RFOs in the phloem sap may be a distinct strategy used by cucumber to relieve the effects of abiotic stresses. The results revealed impairments in photosynthesis (Fig. 3e) and a reduced starch level in mesophyll tissues (Fig. 3a), which indicated that additional sugars found in both mesophyll tissues and exported phloem sap may be derived from starch decomposition in source leaves.

**Figure 4:**
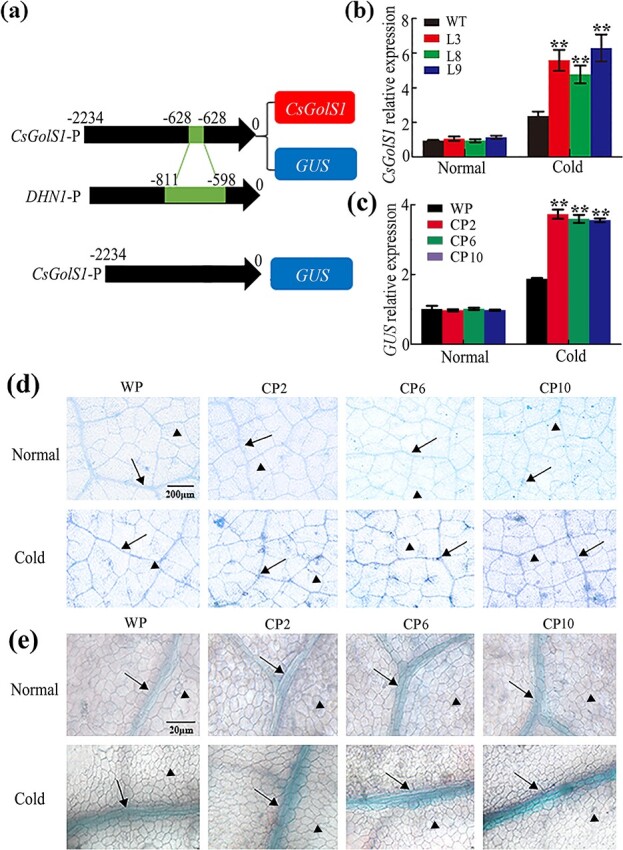
Cold-induced minor vein-specific overexpression of *CsGolS1* and *GUS* was achieved by inserting the tandem tetrameric element LTRE into the *CsGolS1* promoter. WT and T2 seedlings at the five-leaf stage were used*.***a** Diagram of the constructs of the *CsGolS1* promoter inserted with the tandem tetrameric element LTRE that were used for transformation. **b** Expression of *CsGolS1* in source leaves of WT and transgenic lines under normal temperature and cold stress. L, chimera promoter + *CsGolS1*; L3, L8, and L9: three independent transgenic lines. **c** Expression of *GUS* in the WP (WT promoter + *GUS*) and CP (chimera promoter + *GUS*) lines under normal temperature and cold stress. CP2, CP6, and CP10 are three independent transgenic lines. **d**, **e** GUS staining patterns in source leaves from the WP and CP lines under normal temperature and cold stress. The arrows indicate the minor vein and the arrowheads indicate mesophyll cells. The data are shown as mean ± standard deviation (*n* = 5). The asterisks indicate significant differences between the WT and transgenic lines in (**b**) or between the WP and CP lines in (**c**) (Duncan’s test, ^**^P < .01). The scale bars represent 100 μm in (**d**) and 20 μm in (**e**).

**Figure 5:**
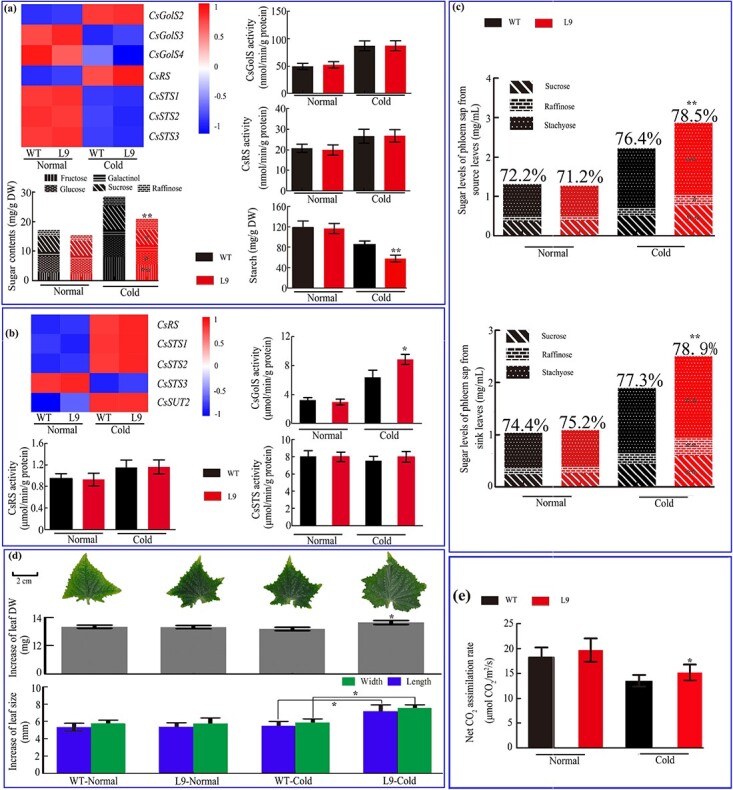
Cold-induced minor vein-specific overexpression of *CsGolS1* promoted sink leaf growth and source leaf photosynthesis by increasing the RFO concentration in the phloem sap and reducing the sugar levels in the mesophyll tissues of the source leaf (L9). Seedlings at the five-leaf stage were used. **a** Mesophyll tissues (source leaf). **b** Vascular tissues (source leaf). **c** Phloem sap; the percentages on the columns indicate the ratio of RFOs to total sugars. **d** Increases in the dry weight (DW) and size of sink leaves after 0–36 hours of exposure to cold stress. **e** Net CO_2_ assimilation rate of source leaves. Samples were collected or measured at 36 hours after cold treatment. L9, transgenic line 9. Enzyme activity is expressed as micromoles of product formed per minute per gram of protein, and the sugar contents estimated with the HPLC method are shown based on dry weight. The data are shown as the mean ± standard deviation (*n* = 5 in **a**–**c** and *n* = 15 in **d** and **e**); in heatmaps, the relative expression level and absolute expression in WT of each gene was used for normalization; the value legend indicates the fold difference in gene expression. The asterisks indicate significant differences between WT and L9 (Duncan’s test, ^*^P < .05, ^**^P < .01).

### Minor vein-specific overexpression confirms the dual role of *CsGolS1* under cold stress

Determination of the function of IC-specific *GolS*, i.e. *CsGolS1* in the stress response would be of particular importance. Among the four types of stresses investigated in this study, cold stress was previously shown to reduce the assimilate translocation velocity [32–34]. Thus, cold stress was selected for further experiments because the observed improvements or lack of change in the assimilate translocation efficiency could be explained by the enhanced phloem sap concentration under cold stress conditions. To further confirm whether *CsGolS1* is responsible for the increase in RFOs in the phloem and whether it plays a role in adaptation under cold stress, a 213-bp cold-induced *cis*-regulatory sequence containing tandem tetrameric low-temperature-responsive elements (LTREs) [35] was inserted into the *CsGolS1* promoter region to achieve cold-induced minor vein-specific overexpression of *CsGolS1*. The structure of the fragment for transformation is shown in Fig. 4a. We obtained five independent transgenic lines (Supplementary Fig. S3) and selected three lines (L3, L8, and L9) for further analysis. As shown in Fig. 4b, similar expression levels of *CsGolS1* were found in the wild-type (WT) and transformed plants under normal temperature. Cold treatment enhanced *CsGolS1* expression in the leaves of all cucumber plants, and this upregulation was more pronounced in the transformed lines than in the WT plants, which indicated that the tandem tetrameric LTRE repeats inserted in the *CsGolS1* promoter significantly increased gene expression under cold stress. We further transformed the chimera promoter + *GUS* structure into the cucumber genome, and the WT promoter + GUS structure was used as a control (Fig. 4a). Four independent transformants were obtained with the WT promoter + *GUS* and chimera promoter + *GUS* (Supplementary Fig. S3), and among these only one WT promoter + *GUS* transformant and three chimera promoter + *GUS* transformants are shown in Fig. 4c–e. The expression level and signal strength of GUS staining further confirmed the effect of low temperature and the cold-induced *cis*-element on gene expression (Fig. 4c), and GUS signal localization also revealed that gene upregulation only occurred in vascular tissues (Fig. 4d and e).

**Figure 6:**
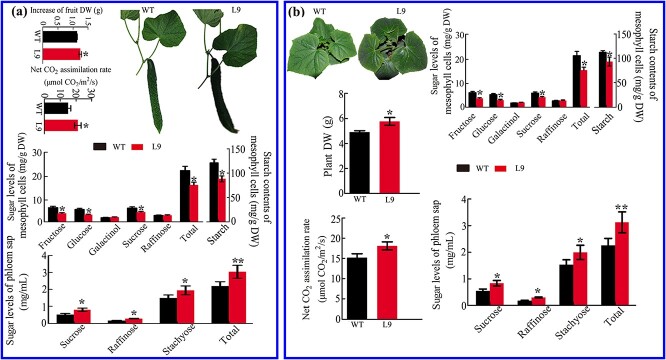
Cold-induced intermediary cell-specific overexpression of *CsGolS1* promoted fruit growth, whole plant development, and source leaf photosynthesis under cold stress. **a** Cucumber plants at the 20-node stage; one fruit between the 10th and 12th nodes (from the bottom) remained. Cold treatment was applied, and the increase in the fruit dry weight (DW) from 8 to 10 days after anthesis was measured. Source leaf samples were collected or measured at 4.00 pm on the second day of the treatment. **b** Cucumber seedlings obtained 35 days after germination were grown under normal or low temperature starting after germination. Source leaf samples were collected or measured at 4.00 p.m. on the 35th day after germination. The data are shown as the mean ± standard deviation (*n* = 5 for the sugar measurements and *n* = 15 for leaf growth, plant dry matter accumulation, and net CO_2_ assimilation rate measurements). The asterisks indicate significant differences between WT and L9 (Duncan’s test, ^*^P < .05, ^**^P < .01).

As shown in Fig. 5, under normal conditions the WT and transgenic seedlings (five-leaf stage) showed similar gene expression, enzyme activity, and sugar contents in mesophyll tissues, vascular tissues, and phloem sap. After cold treatment, minor vein-specific overexpression of *CsGolS1* exerted no obvious effect on the expression and enzyme activity of other genes in either mesophyll or vascular tissues. However, the enhanced expression of *CsGolS1* specifically in the minor vein significantly increased the RFO level and the ratio of RFOs to total sugars in the petiole phloem sap of both source and sink leaves (Fig. 5c). Under cold stress the transgenic plants exhibited a higher growth rate of sink leaves than WT plants (Fig. 5d). Moreover, the *CsGolS1*-overexpressing plants presented lower sucrose, glucose, fructose, and starch contents in mesophyll cells (Fig. 5a) and a higher CO_2_ assimilation rate in source leaves compared with the WT seedlings (Fig. 5e). Because the assimilate translocation velocity is always slower under cold stress than under normal temperature [32] and because the cross-sectional area of the phloem is unlikely to be enlarged by transformation, these results suggest that minor vein-specific overexpression of *CsGolS1* resulted in an enhanced RFO concentration in the phloem sap, which improved the assimilate translocation efficiency and accelerated sink leaf growth under cold stress. The increased RFOs in the phloem sap may be derived from soluble sugars and starch in source leaves under this condition. The reduced sucrose or hexose levels in source leaves may alleviate the feedback inhibition of photosynthesis.

To test whether this promotion effect on sink tissue growth under cold stress also occurs in fruits, one fruit between the 10th and 12th nodes (from the bottom) was retained, and cold treatment was applied from 8 to 10 days after anthesis. As expected, L9 plants exhibited faster fruit growth than the WT plants (Fig. 6a). We further investigated the effect of the minor vein-specific overexpression of *CsGolS1* on whole plant growth under cold stress. As shown in Fig. 6b, L9 plants accumulated more dry matter than WT plants. Further investigation revealed that these faster growth rates were associated with higher concentrations of sugars in the phloem, lower sucrose, hexose, and starch levels in mesophyll tissues, and higher photosynthetic activity in source leaves (Fig. 6). Similar results were observed in another transformed line, L3 (Supplementary Fig. S4). We further planted WT and L9 cucumber plants in the cold season (spring 2020) and found that L9 plants exhibited more vigorous growth than WT plants (Supplementary Fig. S5).

## Discussion

Multiple isoforms of *GolS* in the same plant display different organ-specific expression patterns, which is considered evidence of the functional diversity of these glycosyltransferases. In apple, *MdGolS1*, *3*, and *4* were mainly expressed in mature seeds, whereas *MdGolS2* showed higher mRNA concentrations in dormant buds [5]. In cassava, *MeGolS1*, *3*, *4*, *5*, and *6* were highly expressed in leaves and midveins and *MeGolS3*, *4*, *5*, and *6* exhibited abundant accumulation in fibrous roots [6]. In RFO-translocating plants, the most important information on the IC-specific expression pattern of *GolS* was obtained from melon. Volk *et al*. [36] found that both *CmGolS1* and *CmGolS2* are highly expressed in melon mature leaves, and the low abundance of these two mRNAs in mesophyll tissues indicate that they are primarily expressed in vascular tissues. Haritatos *et al*. [11] expressed *GUS* directed by the *CmGolS1* promoter in *Arabidopsis* and cultivated tobacco and mainly detected the signals in the minor veins of mature leaves. Both of these plants are sucrose-translocating species, which suggests that the IC-specific regulatory mechanism of the *CmGolS1* promoter is highly conserved across the plant kingdom. Truncation and mutagenesis analysis of the promoter region identified three closely arranged sequences that modulate this tissue specificity [37]. To further determine whether a similar IC-specific regulatory mechanism exists in other cucurbits, 44 putative GolSs from Cucurbitaceae and their promoter regions were collected for phylogenetic analysis (Supplementary Fig. S1, Supplementary Tables S2 and S3). We found that all cucurbit species analyzed in this study have one isoform of *GolS* with three blocks in the promoter region, which suggests that this IC-specific regulatory mechanism may be conserved in Cucurbitaceae. To ascertain whether other RFO-translocating species also adopt these sequences to acquire the IC specificity of certain *GolS*s, we collected all 12 putative *GolS* genomic DNAs from *Olea europaea* (a RFO-translocating species [38]). However, no homologous sequence of three conserved cascades was found in the promoter regions of all 12 *OeGolS*s (Supplementary Table S4), which indicates that the regulatory program specific to ICs may differ between Cucurbitaceae and other families. According to our data, *CsGolS1* is localized in the minor vein, whereas the other three isoforms are mainly expressed in mesophyll cells. The data shown in Fig. 1 indicate that *CsGolS2*, *3*, and *4* are responsible for raffinose biosynthesis in mesophyll tissues under various stress conditions.

The role of raffinose in abiotic stress tolerance is well established, and this trisaccharide may act as an osmotic regulator or an ROS scavenger [39, 40]. However, the function of stachyose in the environmental stress response depends on the plant species. In *Arabidopsis*, stachyose biosynthesis is not induced by salt, cold, or heat stress [3, 41], whereas in the RFO-translocating species *Ajuga reptans* stachyose accumulates in winter leaves and is thought to exert protective effects on vacuolar membranes [42]. The current study revealed that the mesophyll tissues of stressed cucumber seedlings exhibit the accumulation of raffinose but not stachyose, which indicates that raffinose, but not stachyose, plays protective roles in cucumber seedlings similar to those in *Arabidopsis*. However, Lü *et al*. [26] reported that cold stress upregulates *CsSTS* expression and increases stachyose synthase (STS) activity and stachyose levels in cucumber leaves, although the exact tissue in which stachyose accumulates was not identified in this study. In our study, stachyose and CsSTS activity was primarily found in vascular tissues; thus, the role of stachyose enrichment reported by Lü *et al*. [26] may be similar to that elucidated in our study (accelerating assimilate translocation). The tissue-specific localization of raffinose and stachyose and their biosynthesis have been studied in several cucurbit species. Raffinose and stachyose dominantly exist in vascular tissues [26]. However, some research groups have provided evidence that raffinose and stachyose are also synthesized in mesophyll tissue [21, 43–45]. In this study, relatively low but notable raffinose synthase (RS) activities were found in cucumber mesophyll cells (Fig. 3), which suggests that raffinose could be synthesized in this tissue. In another study conducted at our laboratory (Dai *et al.*, unpublished work), we observed low accumulation of stachyose in mesophyll tissues of cucumber mature leaves. Multiple isoforms of catabolic enzymes of RFOs are found in cucumber mesophyll cells [27]. Thus, the lack of stachyose in mesophyll tissues observed in the current study may be due to the rapid catabolism of this tetrasaccharide in cucumber seedling leaves.

Multiple nutritive and signal compounds, including proteins, small peptides, various RNAs, phytohormones, amino acids, mineral elements, and carbohydrates, are translocated in the phloem sap of plants [23]. The phloem sap composition is influenced by environmental factors, biotic stresses, genotypes, and cultural practices, which exert pronounced effects on plant development. Ogden *et al*. [46] observed 169 proteins that exhibit significant changes in abundance within tomato phloem sap during periods of drought and recovery. Jakobs and Müller [47] found that aphid infestation of *Tanacetum vulgare* leads to changes in phloem sap chemistry. Tietel *et al*. [48] reported that the phloem sap composition of mandarins (*Citrus reticulata*) is directly affected by rootstock and scion interactions. In our study, different stresses increased both the total soluble sugar concentrations and the ratios of RFOs to total sugars, and the minor vein-specific overexpression experiment suggested that these increases may constitute a distinct mechanism used by cucumber to accelerate assimilate translocation under cold stress. A similar phenomenon was also detected in melon [32]. However, Gil *et al*. [49] reported that *CmGolS1* is not induced and that the ratio of RFOs to sucrose in melon plants decreases in response to cucumber mosaic virus (CMV) infection and high-temperature treatment. We noticed that the authors selected the youngest leaf from 2-week-old cucumber seedlings, and the phloem sap sample was collected after 15 days of heat treatment. These differences may account for the discrepancy in the results between the two experiments.

**Figure 7:**
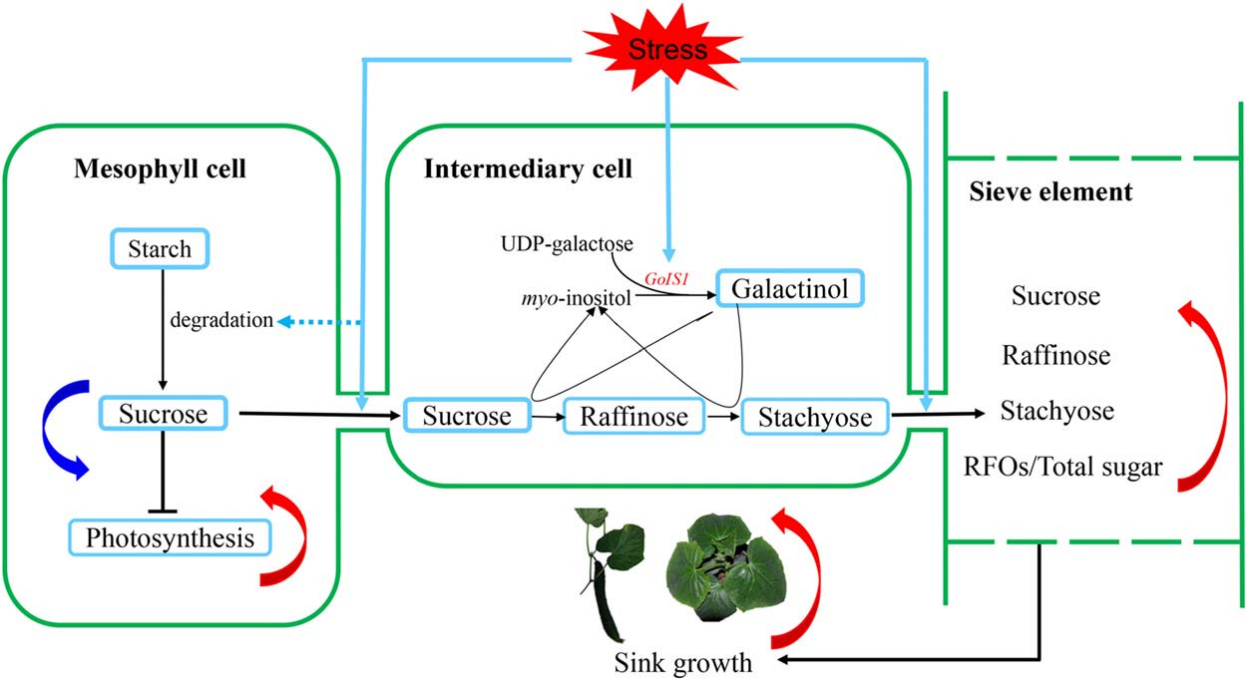
A proposed model of the role of *CsGolS1* in the stress adaptation of cucumber. In cucumber mature leaves, *CsGolS1* is expressed specifically in ICs. Several abiotic stresses, such as cold, can induce the vascular-specific upregulation of *CsGolS1*, which leads to increased RFO biosynthesis in ICs. As a result, stress conditions decrease the sucrose level in mesophyll cells, and this decrease is accompanied by improvements in starch degradation and photosynthesis. In contrast, the total sugar content and the ratio of RFOs to total sugars are increased in sieve tubes, which facilitates assimilate export from leaves and import into sink tissues. Curved arrows: blue, downregulated; red, upregulated.

Hannah *et al*. [50] reported that a small amount of galactinol and raffinose can be translocated in the phloem of potato plants showing *GolS* or *RS* companion cell-specific overexpression. However, in our study the minor vein-specific overexpression of *CsGolS1* did not affect galactinol but induced a significant increase in the RFO level in phloem sap, which indicated that sucrose-translocating and RFO-translocating plants may have different strategies for the retention and retrieval of different sugars in the phloem sap [51]. The overexpression of *GolS* enhances plant tolerance to abiotic stress, as has been observed in several transgenic experiments [9, 39, 52]. In these studies, the overexpression of *GolS* resulted in RFO overaccumulation in plant tissues and then improved their ability to scavenge ROS. Plants overexpressing *GolS* always retain a normal morphology and are considered potential germplasm resources for abiotic resistance breeding [52]. Here, we also found that *GolS*-overexpressing cucumber plants exhibit improved cold tolerance compared with WT seedlings (Figs 5 and 6; Supplementary Fig. S4). However, in our study *GolS* overexpression was achieved in the minor veins of cucumber leaves, and elevated RFO concentrations were identified in the phloem sap rather than mesophyll tissues. The data shown in Figs 5 and 6 reveal that the tolerance mechanism of these minor vein-specific *GolS-*overexpressing plants is distinct from that of constitutively overexpressing transformants, i.e. the transgenic cucumber plants in our study exhibited higher assimilate translocation efficiency and a faster sink growth rate under cold stress. The increased levels of RFOs in the phloem sap may be partly derived from soluble sugars and starch in source leaves, which resulted in lower sucrose and hexose levels and alleviated the feedback inhibition of photosynthesis in source leaves (Fig. 7). Sugars act not only as carbon sources but also as signal substances in plants. Thus, whether changes in the phloem sap composition exert any other effects on sink growth is worth further study. In conclusion, our study elucidates a previously unknown strategy to improve plant abiotic stress tolerance by altering the phloem sap composition and accelerating assimilate translocation.

## Materials and methods

### Plant materials and growth conditions

Cucumber (*C. sativus* L. var. ‘Jinyou 35’, Tianjin Cucumber Institute, China) plant management was performed according to Miao *et al*. [53]. Briefly, the plants were grown in 10 × 10-cm plastic pots (for seedling growth) or 30 × 30-cm plastic pots (for adult plant growth) containing a peat–vermiculite mixture (2:1, v/v) in a growth chamber. The plants were watered once daily and fertilized weekly with Hoagland nutrient solution. The conductivity and water potential of the substrate were set to 2–2.5 ms/cm and − 10 to −5 kPa, respectively. The day/night temperature and relative humidity in the growth chamber were 28/22°C and 70%, respectively, and a light intensity of 700 μmol/m^2^/s was applied for 12 hours per day (8.00 a.m. to 8.00 p.m.).

### Stress treatment and sampling

Seedlings at the five-leaf stage were used for the salt, drought, heat, and cold treatments. For salt stress, 100 ml of 0.1 M NaCl was applied to the plastic pots. For drought stress, watering was controlled, and the treatment was started once the matric potential reached −0.15 MPa. For the cold and heat treatments, the plants were transferred to other chambers in which the day/night temperature was set to 15/5°C and 35/28°C, respectively. The timing and intensity of the stress treatments were determined by several preliminary experiments. The treatments were started at the beginning of the light period. The control plants were maintained in the original chamber throughout the experiment. The second leaf from the plant bottom (source leaf) was sampled after 0, 6, 12, 24, and 48 hours of the treatment. To compare the RFO biosynthesis patterns among different leaf tissues and between WT and transgenic plants, source leaves, sink leaves (first unfolded leaf from the bottom [54]), and phloem sap from the petiole of the source leaf (from the leaf side) and sink leaf (from the stem side) were sampled after 0 and 36 hours of treatment. For the adult plant experiment, WT and transgenic plants with one fruit (between the 10th and 12th node) were subjected to cold treatment (15/5°C; plants subjected to a temperature of 28/22°C were used as controls) for 2 days (9 and 10 days after anthesis), and the fruit, the leaf at the same node as the fruit, and the phloem sap of the leaf were collected. The net CO_2_ assimilation rate of the leaf was measured at 4.00 p.m. on the second day. In the long-term cold stress experiment, the seedlings were grown at 15/5°C or 28/22°C starting after seed germination. The second leaf from the plant bottom (source leaf), the whole plants, and the phloem sap of the source leaf were sampled, and the net CO_2_ assimilation rate of the source leaf was measured at 4.00 p.m. at the 35th day after germination. The samples were frozen in liquid nitrogen immediately after harvest and stored at −80°C.

### Total RNA isolation and expression analysis of *CsRS* and *CsSUT2*

Total RNA was extracted using the TRIzol reagent (Invitrogen, Shanghai, China), and cDNA was synthesized using MultiScribe™ reverse transcriptase (Applied Biosystems). Quantitative real-time RT–PCR was performed using SYBR Premix Ex Taq™ from TaKaRa (China) and a thermocycler (CFX Connect Real-Time System; Bio-Rad, Hercules, CA, USA) according to the following protocol: 2 minutes at 94°C followed by 39 cycles of 94°C for 15 seconds, 60°C for 15 seconds, and 72°C for 30 seconds. The cucumber 18S rRNA gene and elongation factor 1-alpha (GenBank accession numbers AF206894.1 and EF446145) were used for normalization in all analyses. The primers used for this purpose are listed in Supplementary Table S5.

### Expression analysis of *CsSTS* mRNAs and *CsGolS*s

Full-length mRNA sequencing data have revealed that *CsSTS* produces three transcripts through alternative polyadenylation, namely, *CsSTS1*, *2*, and *3* [29]. To investigate the expression patterns of these transcript isoforms, anchored oligo(dT) primers with 3′ ends consisting of three bases complementary to the mRNA sequences were used to determine the levels of *CsSTS1* and *CsSTS2*. The primer specificity was verified using artificial RNAs as templates according to Zhang *et al*. [29]. Absolute quantitative RT–PCR (qRT–PCR) was performed to compare the expression abundance of different isoforms of *CsSTS* and *CsGolS*s. The generation of standard curves and the calculation of copy numbers were conducted according to Zhang *et al*. [29].

### Carbohydrate analysis

Soluble sugars from tissues were extracted and analyzed by HPLC as described by Miao *et al*. [53]. Briefly, the samples were ground, extracted with 80% ethanol, and evaporated to dryness *in vacuo*. The residues were redissolved in distilled water and deionized on Dowex 50 × 8 and Dowex 1 × 8 coupled columns. The column was eluted with distilled water, and the solution was dried and resuspended in distilled water for HPLC analysis. The soluble sugars in the phloem sap were extracted and assayed as described by Mitchell *et al*. [55] with modifications. Briefly, a measured volume of 10 μl of exudate was collected in 20-μl microfuge tubes and transferred to microfuge tubes containing 480 μl of 80% β-mercaptoethanol. The samples were then evaporated at 40°C with a rotary evaporator. After evaporation, the samples were dissolved in 500 μl of ultrapure water and filtered through a 0.22-μm syringe filter for HPLC assay. The soluble sugars were separated on a Waters Sugar-Pak I column at 80°C using water as the mobile phase with a flow rate of 0.6 ml/minute. Stachyose, sucrose, galactinol, glucose, and fructose were identified through comparisons with the retention times of known standard sugars (Sigma), and their levels were quantified using a refractive index detector. The sugar contents in tissues and phloem sap are expressed as milligrams per gram of dry weight and milligrams per milliliter, respectively.

### Enzyme activity determination

The extraction of GolS, RS, and STS and assays of their activities were performed according to our previous works [27, 28], with the exception that enzyme activity was evaluated per gram of protein rather than per gram of fresh weight. The samples used for the enzyme activity assay were extracted using HEPES buffer (50 mM HEPES-NaOH pH 7.4, 5 mM MgCl_2_, 1 mM EDTA, 1 mM EGTA, 0.1% Triton X-100, 10% glycerol, 2 mM benzamidine, 2 mM aminocaproic acid, 1.5 mM PMSF, and 1 g/l PVPP). For the GolS activity assays, the reaction mixture (20 ml) contained 50 mM HEPES-NaOH (pH 7.0), 1 mM dithiothreitol, 5 mM MnCl_2_, 20 mM myoinositol, 5 mM UDP-d-galactose, and 10 ml of the extracts. The reaction mixtures were incubated at 30°C for 15 minutes. For the RS assay, the reaction buffer contained 50 mM HEPES–NaOH (pH 7.0), 1 mM dithiothreitol, 10 mM galactinol, and 40 mM sucrose. The mixtures were incubated at 30°C for 3 hours. The protocol of the STS assay was the same as that of the RS assay, with the exception that galactinol was replaced by raffinose in the reaction system. All reactions were stopped by boiling for 5 minutes. The mixture was centrifuged at 28 000 × g for 5 minutes, and the supernatant was passed through a 0.45-μm filter. All enzyme activities are expressed as micromoles of product formed per minute per gram of protein, the producing sugars were determined by the above-mentioned HPLC methods, and the protein concentration of the samples was determined according to Bradford [56] using bovine serum albumin as the standard.

### Transformation of cucumber

For cucumber transformation, cucumber seeds were soaked in water at room temperature for 4 hours, and the seed coat was then removed. The seeds were surface-sterilized with 70% ethanol for 40 seconds and with 3% sodium hypochlorite solution for 15 minutes and then rinsed six times with sterile water. The sterilized seeds were sown on 1/2 Murashige and Skoog (MS) medium (Solarbio, Beijing, China) at 28–30°C. Two days after germination, both one-quarter proximal and one-quarter distal parts of each cotyledon were removed, and the remaining part was used as the explant. The explants were inoculated with *Agrobacterium* GV3103 suspension harboring the target vectors for 12 minutes. Sterilized filter paper was laid on the MS medium with 50 μmol/l acetosyringone, and the infected explants were cocultivated on the paper for 2 days in darkness at 23°C. After cocultivation, the explants were transferred to differential medium (MS + 3 mg/l 6-benzylaminopurine + 0.05 mg/l 1-naphthylacetic acid + 2 mg/l abscisic acid + 2 mg/l silver nitrate + 100 mg/l kanamycin + 200 mg/l Timentin) for 20–30 days. The emerged shoots were excised and subcultured on elongation medium (MS + 1 mg/l gibberellic acid + 0.1 mg/l 6-benzylaminopurine + 0.01 mg/l 1-naphthylacetic acid + 2 mg/l silver nitrate + 100 mg/l Timentin) for 20–30 days. Putative transformed shoots (at approximately the 2-cm stage) were further cut and transferred to rooting medium (MS + 0.1 mg/l 1-naphthylacetic acid + 200 mg/l Timentin). Putative positive rooting plants (with three or four roots) were planted in the substrate (peat–vermiculite mixture, 2:1, v/v) and moved to a growth chamber for further development (Supplementary Fig. S6). The regenerated plants were screened by PCR for integration of the constructs. The primers used for this purpose are listed in Supplementary Table S5.

### Histochemical localization of *CsGolS*s by GUS assay

Cucumber genomic DNA was extracted from leaves using the DNAquick Plant System (TIANGEN, Beijing, China). Approximately 2 kb before the initiation codon is considered the putative promoter region of certain genes [57], and − 2234/−1853/−1995/−1947 bp to 0 bp before the initiation codon of *CsGolS1*, *CsGolS2*, *CsGolS3*, and *CsGolS4*, respectively, was selected as the promoter region to facilitate primer design. The PCR products were ligated into pCAMBIA2301 vector before the *GUS* coding sequence to obtain the *pCsGolS*::*GUS* fusion constructs using a homologous recombination method (ClonExpress™ II One Step Cloning Kit, Vazyme Biotech, Nanjing, China). The primers used for promoter amplification are listed in Supplementary Table S5. *Agrobacterium*-mediated transformation was performed to transfer the *GUS* reporter constructs into cucumber plants. The tissue-specific localization of the GUS reporter in leaves was investigated by incubating the tissues in GUS staining solution (Solarbio Science & Technology Co., Ltd, Beijing, China) at 37°C overnight. After staining, some leaves were cleaned with 75% ethanol and observed under an anatomical lens (SZX16, Olympus, Japan). Other samples were prepared as paraffin sections according to the standard protocol to observe the anatomical structures of veins and mesophyll tissues [58].

### Separation of the different leaf tissues

Mature cucumber leaves were used to obtain vascular tissues and mesophyll cells with the TAPE sandwich method [59] (Supplementary Fig. S7). Two tape stripes were attached to both sides of the leaf and then separated. The vascular tissue adhered to the adaxial tape. Both the adaxial and abaxial tapes containing mesophyll cells were incubated for 15 minutes in enzymolysis solution [1% w/v cellulase ‘Onozuka’ R10 (Yakult, Tokyo, Japan), 0.25% w/v macerozyme ‘Onozuka’ R10 (Yakult, Tokyo, Japan), 0.4 M mannitol, 10 mM CaCl_2_, 20 mM KCl, 0.1% w/v bovine serum albumin (Sigma-Aldrich, Shanghai China), and 20 mM MES, pH 5.7] under constant agitation at 50 rpm. During this process, spongy mesophyll cells attached to the epidermis were released into the solution. The cells were collected by centrifugation at 200 × g and 4°C for 2 minutes and washed twice with washing buffer (0.4 M mannitol, 15 mM MgCl_2_, 4 mM MES, pH 5.7). The adaxial tape containing vascular tissue was further immersed in cold washing buffer, and the vascular tissue network was removed with tweezers and washed twice in washing buffer. All collected mesophyll cells and vascular tissues were frozen in liquid nitrogen and stored at −80°C until further processing. To verify the efficiency of separation, *CsGAPDH* was used as a mesophyll cell marker [31] and *CsSUT2* was used as a vein marker [24].

### Measurement of the net CO_2_ assimilation rate

The net CO_2_ assimilation rate of source leaves was measured using a gas exchange system (CID-PS CO_2_ Analyzer System, CID, Vancouver, WA, USA). The air temperature, air relative humidity, CO_2_ concentration, and photosynthetic photon flux density were maintained close to the conditions in the above-mentioned climate chamber.

### Phloem sap collection and sink leaf and fruit growth measurement

To evaluate the effect of low temperature on assimilate transport and sink growth, the petiole (of the second leaf from the plant bottom) was cut, and phloem sap was collected with capillary tubes from the leaf side according to Mitchell and Madore [32]. To minimize the effect of contamination from the protoplast of cut surface cells and the xylem sap, the first 2 seconds of sap collection were erased, and the consistency of the watering time and quantity among plants was strictly controlled (except drought treatment). For all the plants, watering was stopped 2 days before sampling. All phloem sap collections were performed within 2 to 60 seconds after cutting, and a similar volume of phloem sap (~10 μl) was collected from all the plants. The length and width of the first unexpanded leaf (from the plant bottom) were measured at the beginning and end of the low-temperature treatment, and phloem sap was also collected from the plant side from the petiole of these sink leaves. The dry weight of leaves, fruits, and whole plants was measured by drying the samples in an oven at 60°C to a constant weight.

### Cold-induced minor vein-specific overexpression of *CsGolS1* and *GUS*

To realize the cold-induced minor vein-specific overexpression of *CsGolS1*, a 213-bp DNA fragment containing tandem LTREs [35] from the cucumber dehydrin gene (XM_011659051) promoter (−811 bp to −598 bp) was inserted into the promoter region of *CsGolS1* at −628 bp. The fragment was amplified using homologous recombination adapter-containing primers. Simultaneously, the fragments of the *CsGolS1* genome sequence from −2234 bp to −628 bp and from −628 bp to +2129 bp were also amplified using primers containing homologous recombination adapters. The above-mentioned primers used for amplification are listed in Supplementary Table S5. Three fragments were ligated, and the ligated fragment was further ligated into the pCAMBIA2301 vector digested with PmaC I and Pst I.

To achieve cold-induced minor vein-specific overexpression of *GUS*, fragments of the *CsGolS1* genome sequence from −2234 bp to −628 bp and from −628 bp to 0 bp were also amplified using primers containing homologous recombination adapters. The two fragments and the 213-bp DNA fragment were ligated, and the long fragment was inserted before 5′ *GUS* in the pCAMBIA2301 vector digested with PmaC I and Eam1105 I. Moreover, fragments of the *CsGolS1* genome sequence from −2234 bp to 0 bp were amplified using primers containing homologous recombination adapters. The fragment was inserted before 5′ *GUS* in the pCAMBIA 2301 vector using a fragment digested with PmaC I and Eam1105I as a negative control. A homologous recombination cloning strategy was adopted to construct all above-mentioned vectors using the ClonExpress™ II One Step Cloning Kit (Vazyme Biotech, Nanjing, China).

### Statistical analysis

The measurements of gene expression, enzyme activity, and sugar contents were repeated five times and the growth assays of leaves, fruits, and seedlings were conducted 15 times. SPSS version 15.0 (SPSS, Inc., Chicago, IL, USA) was used for one-way ANOVA of all data. The differences among the treated samples were evaluated at the 0.05 and 0.01 probability levels using Duncan’s test.

## Acknowledgements

This research was supported by the National Key Research and Development Program (2018YFD1000800) and the National Natural Science Foundation of China (31872107, 31672160 and 32072579).

## Author contributions

M.M.M. designed the experiments and coordinated the project. H.B.D., Z.H.Z., and Z.G.W. performed the experiments, analyzed the data, and drew conclusions based on the results. H.B.D. wrote the manuscript. Z.P.Z. and W.W.K. helped perform the analysis with constructive discussions. All authors read and approved the final manuscript.

## Data availability

All raw data underlying the results presented here are available on request.

## Conflict of interest

The authors declare that they have no conflicts of interest.

## Supplementary data

Supplementary data is available at *Horticulture Research* online.

## Supplementary Material

Web_Material_uhab063
