# Supplementary material for: Galactinol synthase 1 improves cucumber performance under cold stress by enhancing assimilate translocation
Source: Hortic Res. 2022 Jan 20;9:uhab063. doi: 10.1093/hr/uhab063 (PMC9015895; doi:10.1093/hr/uhab063)
Supplement: Web_Material_uhab063 [file web_material_uhab063.zip › Supplementary material (figure S1 S2 S3 S4 S5 S6 S7 table S2 S4 S5).doc]

**Supporting information**

**Fig. S1** Phylogenetic analysis of Cucurbitaceae GolSs. (a) Amino acid sequences; (b) Promoter sequences. Detailed information on the selected Cucurbitaceae *GolS* genes is listed in Table S1.

**Fig. S2** PCR identification (a) and selective GUS histochemical assay (b) of transgenic lines transformed by *CaMV35s*::*GUS* or *pCsGolSs*::*GUS* constructs. The number above lanes in (a) and in (b) represent independent transformants. Arrows indicate the minor vein and arrowheads indicate the mesophyll cell. Scale bars is 20 μm in (b).

**Fig. S3** PCR identification (a) and selective GUS histochemical assay (b) of transgenic lines transformed with cold-induced minor vein-specific overexpression constructs. The number above lanes in (a) represent independent transformants. GUS staining patterns of three independent transformants of WP were shown in (b). L: chimera promoter+*GolS1*, WP: WT promoter+*GUS*, CP: chimera promoter+*GUS.* The structure of the chimera promoter is shown in Fig 4. Arrows indicate the minor vein and arrowheads indicate the mesophyll cell. Scale bars is 20 μm in (b).

**Fig. S4** Cold-induced intermediary cell-specific overexpression of *CsGolS1* promoted fruit growth, whole plant development and source leaf photosynthesis under cold stress. (a) Cucumber plants at the 20-node stage; one fruit between the 10^th^ and 12^th^ nodes (from the bottom) was remained. Cold treatment was carried out, and the increase in fruit dry weight was measured from 8 to 10 days after anthesis. Source leaf samples were collected or measured at 4:00 pm on the second day of the treatment. (b) Cucumber seedlings 35 d after germination, seedlings were grown under normal or low temperature starting from germination. Source leaf samples were collected or measured at 4:00 pm on the 35^th^ d after germination. Data are the means ± SD (n = 5 for sugar measurements and n=15 for leaf growth, plant dry matter accumulation and net CO_2_ assimilation rate measurements). The asterisks indicate significant differences between the WT and L3 (Duncan’s test, * P < 0.05 and ** P < 0.01).

**Fig. S5** Transgenic (L9) and wild type (WT) cucumber plants in the field. Seeds were sowed at Jun 15, 2020 and planted in Feb 20, 2020 in a plastic tunnel. The pictures were taken at March 20, 2020 (a) and Apr 10, 2020 (b). The temperature in the tunnel during March and April was about 15-23 °C/9-13 °C (day/night).

**Fig. S6** Genetic transformation of cucumber. (a) Steriled seeds. (b) Co-cultivation. (c) A regenerated Bud. (d) Shoot elongation. (e) Rooting. (f) Domestication.

**Fig. S7** Physical separation of mesophyll and vascular tissues of cucumber leaves. (a) Upper epidermis was peeled away from leaf disks. A1: Two tape stripes were attached to both sides of the leaf, A2: The mesophyll and vascular tissue was adhering to the adaxial tape, A3: Tapes containing epidermis. (b) Tissues of A2 in the enzymolysis solution. (c) Removal of debris using a 30 µm cell strainer. (d) Centrifuged mesophyll cells. (e) Tissues of A2 in enzyme solution after sonication follows (a). (f) Isolation of vasculature tissues from other tissues. (g) Separated vascular tissues, scale bars is 100 μm.

**Table S2** Information on GolS proteins collected from Cucurbitaceae in this study.

| Gene Name | Accession Number in NCBI | Accession Number in CuGenDB | The length of coding sequences | Molecular weight (MW)/Da | Theoretical isoelectric point | Linkage group distribution |
| --- | --- | --- | --- | --- | --- | --- |
| *CsGolS1* | AY237112 |  | 331aa | 38261.79 | 4.82 | 6 |
| *CsGolS2* | XM_004150790 |  | 336aa | 38448 | 5.4 | 3 |
| *CsGolS3* | XM_004139476 |  | 326aa | 37830.41 | 5.51 | 1 |
| *CsGolS4* | XM_004135204 |  | 332aa | 37916.52 | 5.43 | 5 |
| *CmGolS1* | XM_008460096.2 |  | 331aa | 38291.77 | 4.78 | 11 |
| *CmGolS2* | AY077641.1 |  | 332aa | 37937.53 | 5.59 | 10 |
| *CmGolS3* | XM_008465371.2 |  | 325aa | 37792.36 | 5.6 | 12 |
| *CmGolS4* | XM_008449238.2 |  | 335aa | 38512.01 | 4.97 | 14 |
| *ClGolS1* | AY379777.1 |  | 331aa | 38346.77 | 4.76 | 6 |
| *ClGolS2* |  | Cla010080 | 338aa | 38236.72 | 5.24 | 5 |
| *ClGolS3* |  | Cla010955 | 320aa | 37190.64 | 5.72 | 1 |
| *ClGolS4* |  | Cla017336 | 337aa | 38657.39 | 5.5 | 10 |
| *CmaGolS1* |  | CmaCh17G003900 | 331aa | 38371.86 | 4.73 | 17 |
| *CmaGolS2* |  | CmaCh10G011710 | 325aa | 37323.74 | 5.43 | 10 |
| *CmaGolS3* |  | CmaCh18G007950 | 337aa | 38149.57 | 5.52 | 18 |
| *CmaGolS4* |  | CmaCh14G009492 | 340aa | 38783.42 | 5.69 | 14 |
| *CmaGolS5* |  | CmaCh08G012960 | 330aa | 38177.71 | 4.78 | 8 |
| *CmoGolS1* | XM_023102985.1 |  | 331aa | 38325.79 | 4.73 | 17 |
| *CmoGolS2* | XM_023105710.1 |  | 325aa | 37446.76 | 5.11 | 10 |
| *CmoGolS3* | XM_023089030.1 |  | 335aa | 38014.41 | 5.55 | 18 |
| *CmoGolS4* | XM_023079909.1 |  | 342aa | 38975.41 | 4.95 | 14 |
| *CmoGolS5* | XM_023074586.1 |  | 330aa | 38177.71 | 4.78 | 8 |
| *CpGolS1* | XM_023692144.1 |  | 331aa | 38294.73 | 4.76 | 12 |
| *CpGolS2* | XM_023671784.1 |  | 348aa | 39416.16 | 5.15 | 3 |
| *CpGolS3* | XM_023685397.1 |  | 334aa | 37847.28 | 5.57 | 9 |
| *CpGolS4* | XM_023659851.1 |  | 325aa | 37325.67 | 5.26 | 8 |
| *CpGolS5* | XM_023658530.1 |  | 330aa | 38177.71 | 4.78 | 17 |
| *CarGolS1* |  | Carg05567 | 331aa | 38311.76 | 4.72 |  |
| *CarGolS2* |  | Carg11175 | 342aa | 38936.37 | 4.95 |  |
| *CarGolS3* |  | Carg27417 | 325aa | 37411.76 | 5.18 |  |
| *CarGolS4* |  | Carg14473 | 335aa | 38111.57 | 5.65 |  |
| *CarGolS5* |  | Carg18903 | 590aa | 67312.96 | 5.27 |  |
| *McGolS1* | XM_022282702.1 |  | 330aa | 38200.58 | 4.71 |  |
| *McGolS2* | XM_022303831.1 |  | 329aa | 38392.93 | 5.85 |  |
| *McGolS3* | XM_022301321.1 |  | 334aa | 37840.4 | 5.4 |  |
| *McGolS4* | XM_022295421.1 |  | 332aa | 37557.14 | 5.64 |  |
| *LsGolS1* |  | Lsi03G011340 | 1122aa | 125617.63 | 5.52 | 3 |
| *LsGolS2* |  | Lsi04G005550 | 336aa | 38173.71 | 5.43 | 4 |
| *LsGolS3* |  | Lsi09G015120 | 343aa | 39271.16 | 5.53 | 9 |
| *LsGols4* |  | Lsi02G011410 | 273aa | 31891.66 | 5.87 | 2 |
| *BhGolS1* |  | Bhi12M001759 | 331aa | 38385.93 | 4.86 | 12 |
| *BhGolS2* |  | Bhi11M001093 | 327aa | 38537.99 | 5.3 | 11 |
| *BhGolS3* |  | Bhi08M000618 | 321aa | 37268.79 | 5.91 | 8 |
| *BhGolS4* |  | Bhi07M001388 | 341aa | 38503.02 | 5.11 | 7 |

**Table S4** *GolS* in other Families analyzed in this study.

| Species | Gene Name | Accession Number |  |
| --- | --- | --- | --- |
| *Oryza sativa* | *OeGolS3* | [XM_023039934.1](https://www.ncbi.nlm.nih.gov/nuccore/XM_023039934.1) |  |
|  | *OeGolS4* | [XM_023039933.1](https://www.ncbi.nlm.nih.gov/nuccore/XM_023039933.1) |  |
|  | *OeGolS5* | [XM_023035735.1](https://www.ncbi.nlm.nih.gov/nuccore/XM_023035735.1) |  |
|  | *OeGolS6* | [XM_023034724.1](https://www.ncbi.nlm.nih.gov/nuccore/XM_023034724.1) |  |
|  | *OeGolS7* | [XM_023034723.1](https://www.ncbi.nlm.nih.gov/nuccore/XM_023034723.1) |  |
|  | *OeGolS8* | XM_023031891.1 |  |
|  | *OeGolS9* | [XM_023022515.1](https://www.ncbi.nlm.nih.gov/nuccore/XM_023022515.1) |  |
|  | *OeGolS10* | [XM_023021417.1](https://www.ncbi.nlm.nih.gov/nuccore/XM_023021417.1) |  |
|  | *OeGolS11* | [XM_023020193.1](https://www.ncbi.nlm.nih.gov/nuccore/XM_023020193.1) |  |
|  | *OeGolS12* | [XM_023015131.1](https://www.ncbi.nlm.nih.gov/nuccore/XM_023015131.1) |  |
|  | *OsGolS1* | XM_015789894.2 |  |
|  | *OsGolS2* | XM_015773347.2 |  |

| **Table S5** Primers used in the study. | | | |  |
| --- | --- | --- | --- | --- |
| Purpose | | Direction | Sequence of the primer(5' to 3') | Fragment length (bp) |
| Construction of vectors | *CsGolS1*-Promotr | F | cttgcatgcctgcaggtcgacGAATTAAGAGGAGTGTGTGGGTACG |  |
|  |  | R | ttgcgaaggatagtggggTATTGCTTTATTATTCAATTATGGGCC |  |
|  | *CsGolS2*-Prpmoter | F | cttgcatgcctgcaggtcgacTGTCAACTTAGGATAGCCCAAC |  |
|  |  | R | ttgcgaaggatagtggggTTAATCACCGGAGCTACTGC |  |
|  | *CsGolS3*-Promoter | F | cttgcatgcctgcaggtcgacATAAAGACTTATCGAGGTATGATTGGATCT |  |
|  |  | R | ttgcgaaggatagtggggCTCAACAATTCAGGAGCCATGATTACTACA |  |
|  | *CsGolS4*-Promoter | F | cttgcatgcctgcaggtcgacCAACTTCATTGCCATTGCCTTCA |  |
|  |  | R | ttgcgaaggatagtggggTATTCTCAGGAAGTTCAAGAGCC |  |
|  | cucumber dehydrin genome sequence from -811 to -598 | F | atgtaaaaccgaCCATCGGTCTCCTATGGCCTCAT |  |
|  |  | R | AGCCGGTGGTGTCGGTATT |  |
|  | *CsGolS1* genome sequence from -2234 to -628 | F | cctctagagtcgacctgcagGAATTAAGAGGAGTGTGTGGGTACG |  |
|  |  | R | accgatggtcggttttacatAAGGCAGTGTTTGATAATGAT |  |
|  | *CsGolS1* genome sequence from -628 to 0 | F | aataccgacaccaccggctATTGGAGTCCATTTGTTGAGG |  |
|  |  | R | ttctacaggacggacgagtcggttccctcagatctaccatTATTGCTTTATTATTC |  |
|  | *CsGolS1* genome sequence from -2234 to 0 | F | aataccgacaccaccggctATTGGAGTCCATTTGTTGAGG |  |
|  |  | R | ttctacaggacggacgagtcggttccctcagatctaccatTATTGCTTTATTATTC |  |
|  | *CsGolS1* genome sequence from -628 to +2129 | F | aataccgacaccaccggctATTGGAGTCCATTTGTTGAGG |  |
|  |  | R | cctgtaattcacacgtgGGGAAAGGCAGATATAAAACCAGCCTTATTC |  |
| Quantitative real-time PCR | glyceraldehyde-3-phosphate dehydrogenase | F | ATCAACCGCCGCCACTTTG | 300bp |
|  |  | R | TGTCGAGAAGGACAACACCAGGAT |  |
|  | *CsGolS1* | F | GTAGTTGGATTGGCAAAGGGT | 350bp |
|  |  | R | TTGTCAGGGCATTGTTGG |  |
|  | *CsGolS2* | F | GCCTCCGAAAGGTGAAAT | 221bp |
|  |  | R | TCCACGAACTCCCAAATA |  |
|  | *CsGolS3* | F | GATGCTCGTCAAGAAATGG | 283bp |
|  |  | R | CCACAACAAACAATCTCCTC |  |
|  | *CsGolS4* | F | TCGCTCGATTTGAAGGAAGTGG | 112bp |
|  |  | R | GAACATAGGCAATATTGGGCTCAG |  |
|  | *CsRS* | F | ATGAGTATTTTCAGGTTTA |  |
|  |  | R | GACAAAGTCACTATCCCCAGG |  |
|  | *CsSTS* | F | TTGGATTGAAGAAGCTGGTG |  |
|  |  | R | CATGGCTATTCCTTAGCACA |  |
|  | 18s rRNA | F | TCTATAGCCTTGGCCGACAG |  |
|  |  | R | TACAAAGGGCAGGGACGTAG |  |
|  | EF1a | F | CGTTGAGGGTATGAATGTGG |  |
|  |  | R | CCACAATCGGCAATGACAAC |  |
|  | Cucumber sucrose transporter 2 | F | TCTCTTAAGTAATGCTTGCTGCG | 235bp |
|  |  | R | GAGCAGAATCTGATAAGCGTGG |  |
| Reverse transcription primers for *CsSTS*, including two anchor primers | *CsSTS1* | R | TTTTTTTTTTTTTTTTTAAT |  |
|  | *CsSTS2* | R | TTTTTTTTTTTTTTTTCCT |  |
|  | *CsSTS3* | R | TTCCCTCTTTCAAAGGGCAA |  |
| Screening of related transgenic plants | *GUS* | F | CGACGGCCTGTGGGCATTCA | 225bp |
|  |  | R | GCATCGAAACGCAGCACGA |  |
|  | Overexpression of *CsGolS1* | F | ATTGGCTTCGTCTTTGTAGT |  |
|  |  | R | TGTCGGTATTGATTGGATG | 564bp |

Lower case letters indicate recombination adapters.
